# Quantitative washout in patients with hepatocellular carcinoma undergoing TACE: an imaging biomarker for predicting prognosis?

**DOI:** 10.1186/s40644-022-00446-6

**Published:** 2022-01-11

**Authors:** Lukas Müller, Felix Hahn, Florian Jungmann, Aline Mähringer-Kunz, Fabian Stoehr, Moritz C. Halfmann, Daniel Pinto dos Santos, Jan Hinrichs, Timo A. Auer, Christoph Düber, Roman Kloeckner

**Affiliations:** 1 Department of Diagnostic and Interventional Radiology, University Medical Center of the Johannes Gutenberg University Mainz, Langenbeckst. 1, 55131 Mainz, Germany; 2 Department of Radiology, University Hospital Cologne, Cologne, Germany; 3 Department of Diagnostic and Interventional Radiology, University Hospital Frankfurt, Frankfurt, Germany; 4 Department of Diagnostic and Interventional Radiology, Hannover Medical School, Hannover, Germany; 5 Department of Radiology, Charité – University Medicine Berlin, Berlin, Germany

**Keywords:** Hepatocellular carcinoma, Transarterial chemoembolization, Computed tomography, Quantitative washout assessment, Prognosis prediction

## Abstract

**Background:**

The delayed percentage attenuation ratio (DPAR) was recently identified as a novel predictor of an early complete response in patients with hepatocellular carcinoma (HCC) undergoing transarterial chemoembolization (TACE). In this study, we aimed to validate the role of DPAR as a predictive biomarker for short-, mid-, and long-term outcomes after TACE.

**Methods:**

We retrospectively reviewed laboratory and imaging data for 103 treatment-naïve patients undergoing initial TACE treatment at our tertiary care center between January 2016 and November 2020. DPAR and other washin and washout indices were quantified in the triphasic computed tomography performed before the initial TACE. The correlation of DPAR and radiologic response was investigated. Furthermore, the influence of DPAR on the 6-, 12-, 18-, and 24-month survival rates and the median overall survival (OS) was compared to other established washout indices and estimates of tumor burden and remnant liver function.

**Results:**

The DPAR was significantly of the target lesions (TLs) with objective response to TACE after the initial TACE session was significantly higher compared to patients with stable disease (SD) or progressive disease (PD) (125 (IQR 118–134) vs 110 (IQR 103–116), *p* < 0.001). Furthermore, the DPAR was significantly higher in patients who survived the first 6 months after TACE (122 vs. 115, *p* = 0.04). In addition, the number of patients with a DPAR > 120 was significantly higher in this group (*n* = 38 vs. *n* = 8; *p* = 0.03). However, no significant differences were observed in the 12-, 18-, and 24-month survival rates after the initial TACE. Regarding the median OS, no significant difference was observed for patients with a high DPAR compared to those with a low DPAR (18.7 months vs. 12.7 months, *p* = 0.260).

**Conclusions:**

Our results confirm DPAR as the most relevant washout index for predicting the short-term outcome of patients with HCC undergoing TACE. However, DPAR and the other washout indices were not predictive of mid- and long-term outcomes.

**Supplementary Information:**

The online version contains supplementary material available at 10.1186/s40644-022-00446-6.

## Introduction

Hepatocellular carcinoma (HCC) is one of the most common cancers worldwide and greatly contributes to cancer-related deaths [1, 2]. According to the guidelines of the European Association for the Study of the Liver (EASL) and the American Association for the Study of Liver Diseases (AASLD), the Barcelona Clinic Liver Cancer (BCLC) classification system is the preferred framework for predicting prognosis and allocating treatment [3, 4]. For patients with intermediate-stage HCC, the BCLC classification system recommends transarterial chemoembolization (TACE) as the standard of care [5, 6]. However, considerable differences in tumor burden and liver function are observed within the intermediate stage, leading to remarkable heterogeneity in this patient group [7]. Thus, the prediction of prognosis and treatment decision-making remain difficult in these patients [8].

Cross-sectional contrast-enhanced imaging is mandatory for dedicated pre-procedural planning before the initiation of TACE treatment. In addition, imaging plays an essential role in predicting prognosis and in patient stratification. Despite tumor burden, tumor growth rate, and tumor margin, the grade of tumor hypervascularization has been shown to have predictive ability in these patients [9–14]. However, up to 30% of HCCs do not have clear arterial enhancement [15, 16]. Thus, delayed phase washout may be an alternative imaging biomarker for predicting treatment success [17, 18].

In a recent study, Fronda et al. proved not only the important role of washout assessment as a diagnostic tool, but also as a novel and highly predictive imaging biomarker for the early tumor response after TACE [19]. In particular, quantitative assessment of the delayed percentage attenuation ratio (DPAR), defined as the relationship between the attenuation of the adjacent liver parenchyma and the attenuation of the tumor area, was strongly associated with a complete response early after TACE. In particular, a DPAR ≥120 was identified as an optimal cut-off for early response prediction.

We hypothesize that DPAR functions not only as a predictor of the early response, but also as a prognostic factor for short-, mid-, and long-term survival in patients with HCC undergoing TACE. Therefore, the present study evaluated quantitative washout assessment, especially the DPAR, as a novel prognostic imaging biomarker for predicting survival outcome in patients with HCC undergoing TACE.

## Methods

The ethics committee of the Medical Association of Rhineland Palatinate, Mainz, Germany, approved this study (permit number 2021–16013). The requirement for informed consent was waived for the retrospective analysis of clinical data. Patient records and information were anonymized and de-identified prior to analysis. The basis for drafting this manuscript was the TRIPOD guidelines [20].

### Patients

**Fig. 1:**
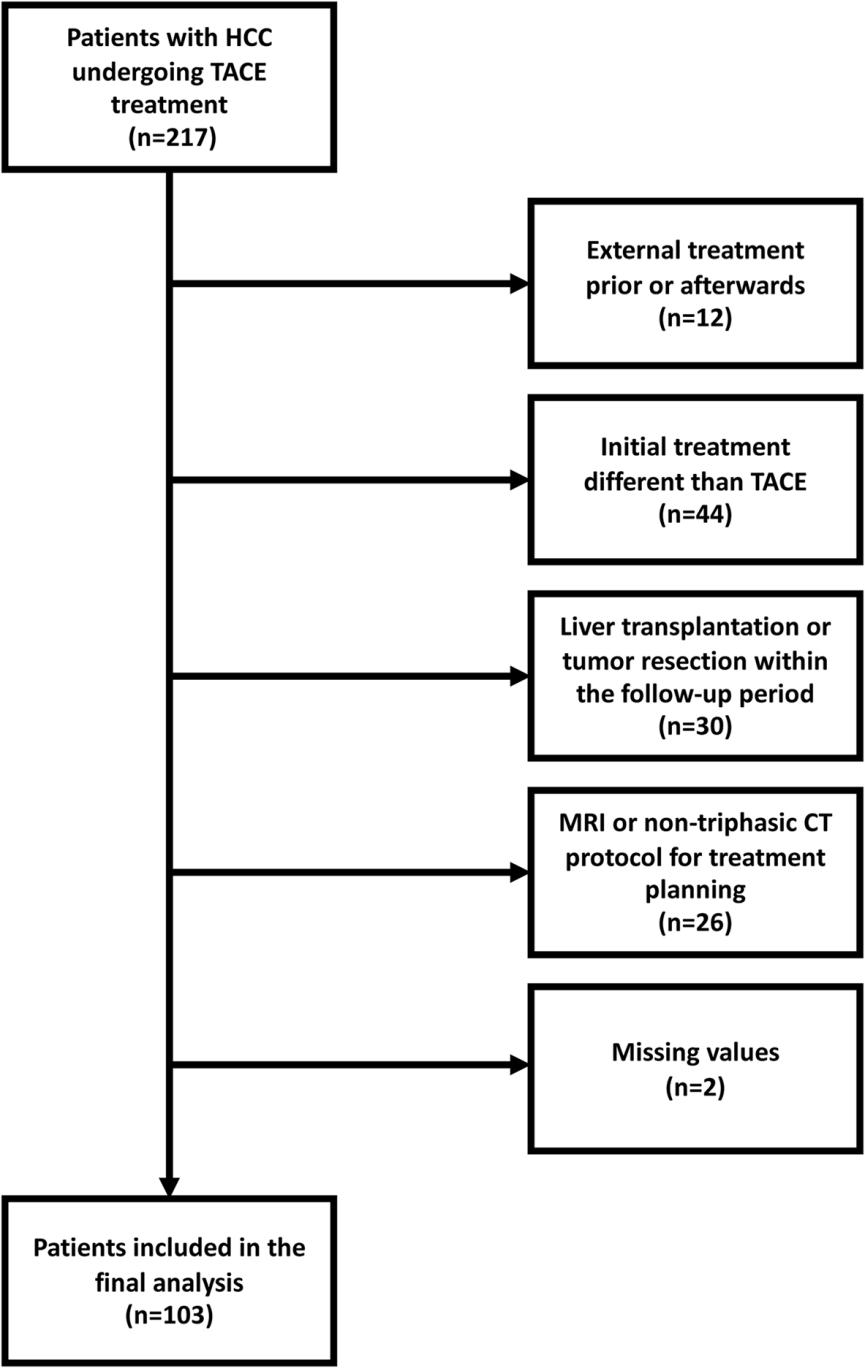
Flowchart of patient inclusion. HCC, hepatocellular carcinoma; TACE, transarterial chemoembolization

Among 217 patients with proven HCC who underwent TACE at our tertiary care center during the inclusion period between January 2016 and November 2020, 114 patients were excluded for the reasons shown in Fig. 1.

### Diagnosis and pre-procedural imaging

HCC was diagnosed using histology or image-derived EASL criteria [3]. All patients underwent a triphasic contrast-enhanced computed tomography (CT) within 1 month prior to their first TACE treatment. All CT images were obtained using a 256- or 64-slice CT scanner (iCT or Brilliance 64, Philips, Eindhoven, the Netherlands). A dedicated imaging protocol was used for HCC diagnosis and follow-up (Table 1). Iomeprol (Imeron 400, Bracco, Milano, Italy) was used as the contrast medium with a body weight-adapted dosage (1.5 mL/kg body weight) and injection rate of 4 mL/s using an automated injector (Accutron CT-D®, Medtron, Germany). Contrast injection was followed by a saline flush with the same flow rate. During post-processing, images with a slice thickness of 1 mm, 3 mm, and 5 mm were reconstructed in the axial orientation and in sagittal and coronal views.

**Table 1:** Scan parameters for contrast-enhanced CT imaging prior to the initial TACE treatment

Parameter	Value
Tube voltage, arterial phase (kVp)	80
Tube voltage, venous phase (kVp)	120
Tube voltage, delayed phase (kVp)	120
Tube current modulation (mA)	120–800
Acquisition slice thickness (mm)	0.625
Detector configuration (rows x mm)	256/64 × 0.625

kVp, kilovoltage peak. mA, milliampere.

### Treatment and follow-up

As reported previously [21], follow-up included clinical examination, blood sampling, and cross-sectional imaging, which was typically repeated every 6 weeks in the case of a viable tumor. In the case of a complete response, this interval was extended to 12 weeks. All patients were extensively discussed in an interdisciplinary tumor board consisting of hepatologists/oncologists, diagnostic and interventional radiologists, visceral surgeons, pathologists, and radiation therapists prior to each treatment decision. TACE was performed in a standardized manner as described in detail elsewhere [22, 23]. The primary endpoint was OS, defined as the time between the initial TACE session and death or last follow-up.

### Image analysis

**Fig. 2:**
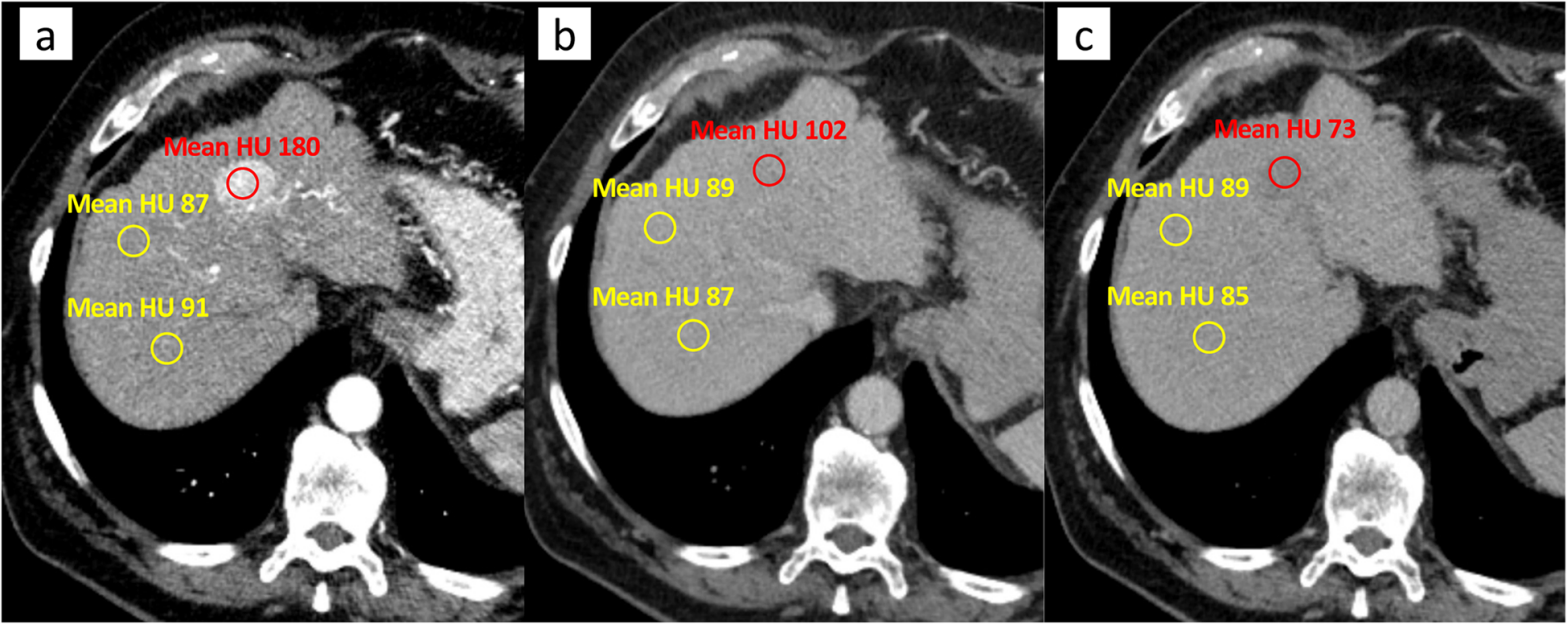
Example of the quantitative image analysis. **A** Arterial (mean hounsefield unit (HU) = 180), (**B**) venous (mean HU = 102), and (**C**) delayed phase (mean HU = 73)

To ensure comparability, the same pre-procedural image analysis was performed as described by Fronda et al. [19]. Briefly, a circular region of interest (ROI) with a diameter of 10–15 mm was drawn manually on the largest tumor area in the arterial phase and delayed phase. For the sake of completeness, we decided to include the venous phase as well. In each phase, two ROIs were drawn in the adjacent hepatic parenchyma, excluding areas with any type of vessels and ducts and areas with image artifacts (Fig. 2).

**Fig. 3:**
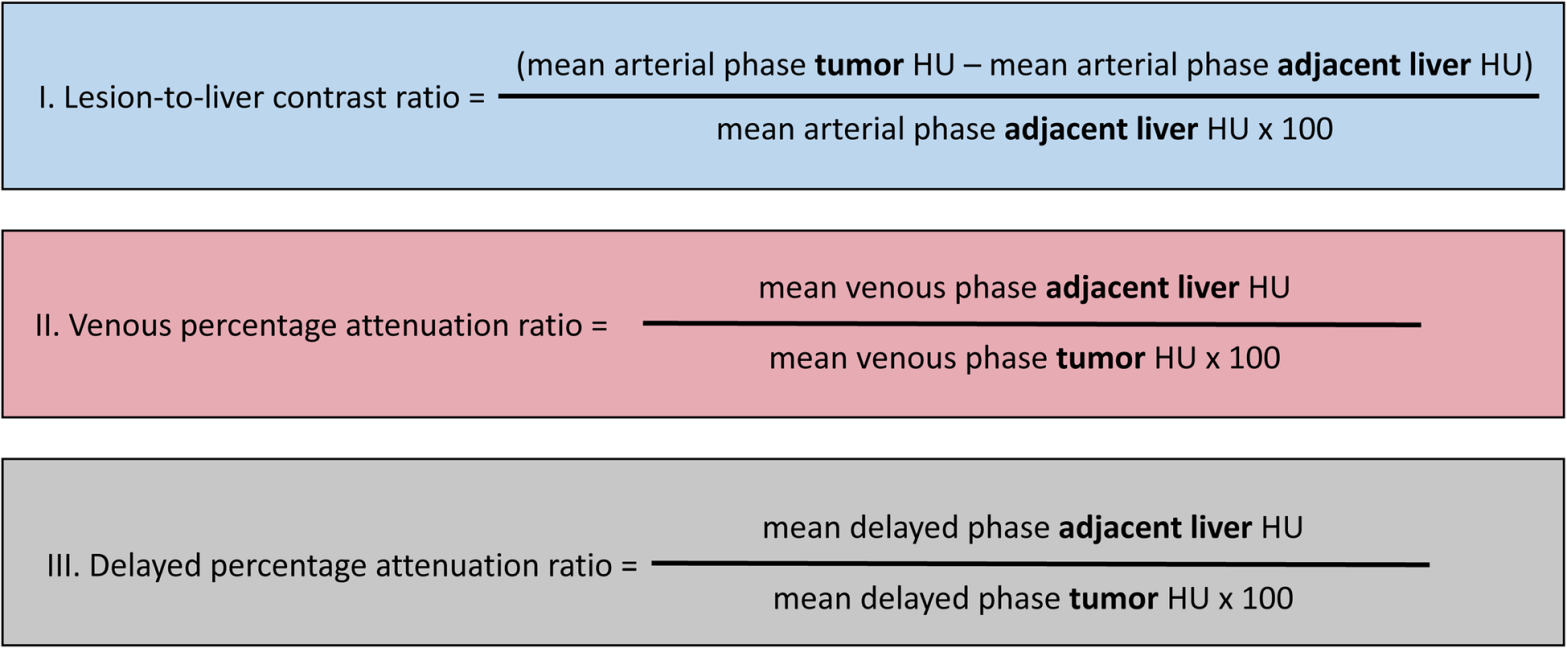
Overview of the calculation of the various wash-in and washout estimates

The following indices for wash-in and washout quantification were calculated as reported previously [19]: lesion-to-liver contrast ratio, venous percentage attenuation ratio, and DPAR (Fig. 3).

We always used the mean attenuation value of the ROI in the tumor area. For the adjacent liver attenuation measurement, we used the average HU value for the two ROIs.

ROIs were drawn by one resident with 3 years of experience in liver imaging and an experienced consultant radiologist with more than 10 years of experience in liver imaging, who were blinded to the survival outcome of the patients.

Radiologic response was assessed using the mRECIST criteria [3, 24].

### Data acquisition

The dataset was acquired from the clinical registry unit (CRU). The CRU is an established registry that prospectively collects information on all patients with liver cancer treated at our tertiary referral center [25]. The database output included demographic data, liver disease status and etiology, laboratory parameters, TACE-related parameters, and information on the tumor burden, including tumor growth pattern, number of lesions, and diameter of the largest target lesion. In the case of missing data, the information was collected from the radiology information system, the hospital information system, or the laboratory database.

### Statistical analysis

Statistical analyses and graphic design were performed in R 4.0.3 (A Language and Environment for Statistical Computing, R Foundation for Statistical Computing, http://www.R-project.org; last accessed 31 072021). Categorical and binary baseline parameters were reported as absolute numbers and percentages. Continuous data were reported as mean, median, range, standard deviation, and median absolute deviation. Standardized cut-offs for the laboratory parameters were derived from our laboratory database. Categorical parameters were compared using Fisher’s exact test and continuous parameters using the Mann-Whitney test. Survival analyses and creation of the Kaplan-Meier curves were performed with the packages “survminer” and “survival” (https://cran.r-project.org/package=survminer, https://CRAN.R-project.org/package=survival, accessed 31 072021). The same packages were used to identify the optimal cut-off value for DPAR. Univariate and multivariate Cox proportional hazards regression models assessing hazard ratios (HRs) and corresponding 95% confidence intervals (CIs) were used to determine the effect of the risk stratification and to evaluate the roles of included factors. *P* < 0.05 was considered significant in all tests.

## Results

### Baseline characteristics

Of the 103 patients included in the retrospective analysis, 92 (89.3%) had liver cirrhosis, with alcohol being the most common etiology. Among the included patients, 25 patients had BCLC stage A, 65 patients BCLC stage B, 6 patients BCLC stage C, and 7 patients BCLC stage D. All baseline characteristics at initial TACE treatment are presented in Table 2.

**Table 2:** Baseline characteristics of the study cohort

Variable	All patients (***n*** = 103)
Median age, years (IQR)	69 (65–76)
Sex, n (%)	
Female	16 (15.5)
Male	87 (84.5)
Etiology, n (%)	
Alcohol	55 (53.4)
Hepatitis B	13 (12.6)
Hepatitis C	5 (4.9)
NASH	9 (8.7)
Hemochromatosis	3 (2.9)
AIH/PBC/PSC	4 (3.9)
Unknown/Other	14 (13.6)
Child-Pugh stage, n (%)	
A	44 (42.7)
B	41 (39.8)
C	7 (6.8)
No cirrhosis	11 (10.7)
BCLC stage, n (%)	
0	0
A	25 (24.3)
B	65 (63.1)
C	6 (5.8)
D	7 (6.8)
Median tumor size of the largest lesion, mm (IQR)	42 (30–60)
Tumor number, n (%)	
Unifocal	19 (18.5)
Multifocal	80 (77.7)
Diffuse growth pattern	4 (3.8)
Laboratory parameters, median (IQR)	
Albumin, g/L	31 (28–36)
Bilirubin, mg/dl	1.44 (0.92–2.5)
Platelet count, per mm^3^	111 (74–172)
AST, U/l	61 (42–88)
ALT, U/l	40 (25–59)
INR	1.2 (1.1–1.3)
AFP, U/l	25 (6–280)
Type of TACE, n (%)	
cTACE	29 (28.2)
DEB-TACE	74 (71.8)

Values are given as n (%) or median (interquartile range) unless otherwise noted. NASH, nonalcoholic steatohepatitis. AIH, autoimmune hepatitis. PBC, primary biliary cholangitis. PSC, primary sclerosing cholangitis BCLC, Barcelona Clinic Liver Cancer. AST, aspartate aminotransferase. ALT, alanine aminotransferase. AFP, alpha fetoprotein. cTACE, conventional transarterial chemoembolization. DEB-TACE, drug-eluting bead transarterial chemoembolization.

### Quantitative imaging analysis

**Fig. 4:**
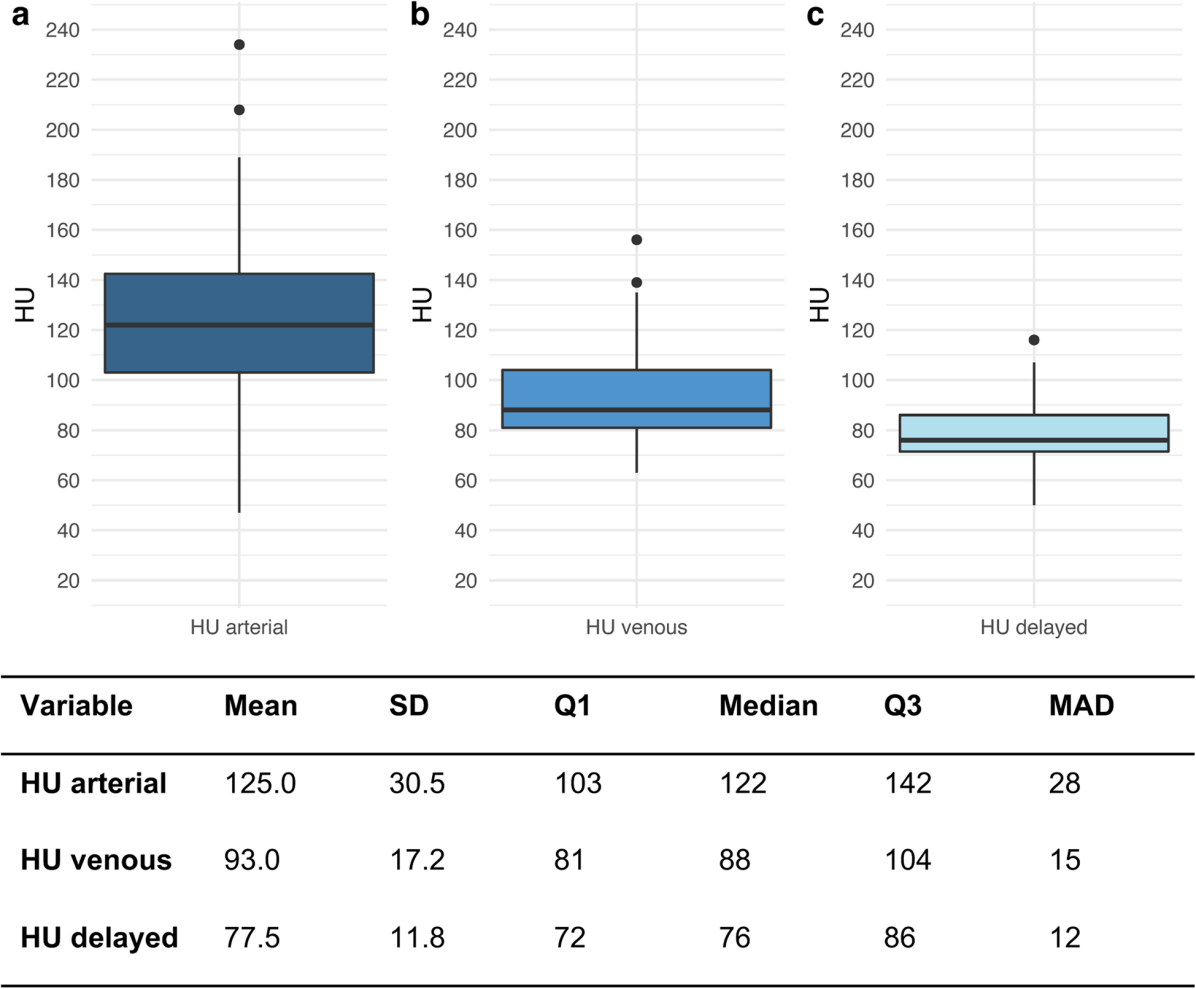
Boxplots of the distribution of attenuation during triphasic CT scan. **A** Arterial, (**B**) venous, and (**C**) delayed. The line indicates the median HU value. The whiskers show the spread of the HU values referenced on the interquartile range. SD, standard deviation. Q1, lower quartile, Q3, upper quartile. MAD, median absolute deviation

Figure 4 shows the distribution of the attenuation values in the tumor tissue during the triphasic CT scan. Standard deviation and median absolute deviation had the largest differences in the arterial phase, whereas the attenuation in the delayed phase had the smallest differences.

For the entire patient cohort, the median (IQR) lesion-to-liver contrast ratio was 41 (26–50), the venous percentage attenuation ratio 109 (101–123), and the DPAR 118 (110–129).

Although the original study by Fronda et al. [19] recommend a placement of just one single ROI in the largest tumor area, we performed a supplementary analysis on the correlation of the attenuation in various tumor regions (Supplementary Fig. 1). Correlation of the attenuation of the largest tumor region and the other tumor region was high for all phases (*p* < 0.001 for all phases). The strongest correlation was observed in the delayed phase.

### Correlation of DPAR and the radiologic response

The DPAR of the target lesions (TLs) in patients with objective response (complete response (CR) or partial response (PR) according to mRECIST) after the initial TACE session was significantly higher compared to patients with stable disease (SD) or progressive disease (PD) (125 (IQR 118–134) vs 110 (IQR 103–116), *p* < 0.001). Furthermore, the number of CR/PR after initial TACE was significantly higher in the group of patients with a DPAR ≥120 than in the group of patients with a DPAR < 120 (*n* = 40 (87.0%) vs *n* = 18 (31.6%), *p* < 0.001). Additionally, the DPAR change was evaluated for 97 patients with a delayed phase CT scan in the first follow-up. The DPAR of patients with an initial value ≥120 showed a mean decrease of 51.2, while the mean decrease in patients with an initial DPAR < 120 was 12.2 (*p* = 0.007). In correlation to the response, patients with CR/PR had a mean DPAR decrease of 54.5, while patients with SD/PD had a mean DPAR increase of 1.4 (*p* < 0.001).

### Correlation of DPAR and the local tumor control rate

The 1-year local tumor control (LTC) rate, defined as CR, PR or SD according to mRECIST [26], was calculated for patients that were alive after twelve months and had follow-up imaging available (*n* = 45). The LTC rate for patients with a DPAR ≥120 was 87.5%, while the LTC rate of patients with a DPAR < 120 was 52.4% (*p* = 0.019). Furthermore, patients with LTC had a significantly higher DPAR (125 (IQR 114–132) vs 112 (IQR 107–118), *p* = 0.029).

### Correlation of DPAR and the number of TACE sessions

The median number of TACE sessions from the baseline for patients with a DPAR ≥120 was 3 (IQR 2–5), while patients with a DPAR < 120 received a median of 2 (IQR 2–4) TACE sessions (*p* = 0.165). After six months, 19 patients (50.0%) with an initial DPAR ≥120 received an additional TACE session and 18 patients (51.4%) of those with an initial DPAR < 120 received an additional TACE session (*p* = 1.000).

### Survival analysis

Table 3 compares the lesion-to-liver contrast ratio and the different washout indices to the survival rates at 6, 12, 18, and 24 months after the initial TACE.

**Table 3:** Comparison of the survival rate for the different imaging indices and time points after the initial TACE

Outcome variable	OS > 6 months (***n*** = 73)	OS ≤ 6 months (***n*** = 30)	***P***-value
Lesion-to-liver contrast ratio	68 (38–100)	81 (26–120)	0.78
Venous percentage attenuation ratio	110 (101–124)	108 (100–122)	0.47
Delayed percentage attenuation ratio	122 (112–131)	115 (106–121)	**0.04**
Delayed percentage attenuation ratio ≥ 120	38 (52.1%)	8 (26.7%)	**0.03**
**Outcome variable**	**OS > 12 months (*n*** **= 40)**	**OS ≤ 12 months (*n*** **= 63)**	**P-value**
Lesion-to-liver contrast ratio	67 (42–87)	69 (36–102)	0.64
Venous percentage attenuation ratio	109 (100–125)	109 (101–123)	0.89
Delayed percentage attenuation ratio	122 (111–131)	117 (110–126)	0.27
Delayed percentage attenuation ratio ≥ 120	22 (55.0%)	24 (38.1%)	0.11
**Outcome variable**	**OS > 18 months (*n*** **= 24)**	**OS ≤ 18 months (*n*** **= 79)**	**P-value**
Lesion-to-liver contrast ratio	70 (54–105)	69 (36–100)	0.59
Venous percentage attenuation ratio	106 (98–118)	110 (102–123)	0.26
Delayed percentage attenuation ratio	122 (113–131)	118 (110–126)	0.31
Delayed percentage attenuation ratio ≥ 120	14 (58.3%)	32 (40.5%)	0.16
**Outcome variable**	**OS > 24 months (*n*** **= 12)**	**OS ≤ 24 months (*n*** **= 91)**	**P-value**
Lesion-to-liver contrast ratio	72 (60–102)	69 (36–100)	0.38
Venous percentage attenuation ratio	100 (97–111)	110 (102–124)	0.05
Delayed percentage attenuation ratio	118 (111–131)	118 (110–128)	0.94
Delayed percentage attenuation ratio ≥ 120	6 (50.0%)	40 (44.0%)	0.76

OS, overall survival.

The DPAR was significantly higher for the patients who survived the first 6 months after TACE, and the number of patients with a DPAR > 120 significantly higher, compared to those who did not survive the first 6 months. However, no significant differences were observed at the other investigated time points. None of the other indices showed any significant difference at any investigated time point.

**Fig. 5:**
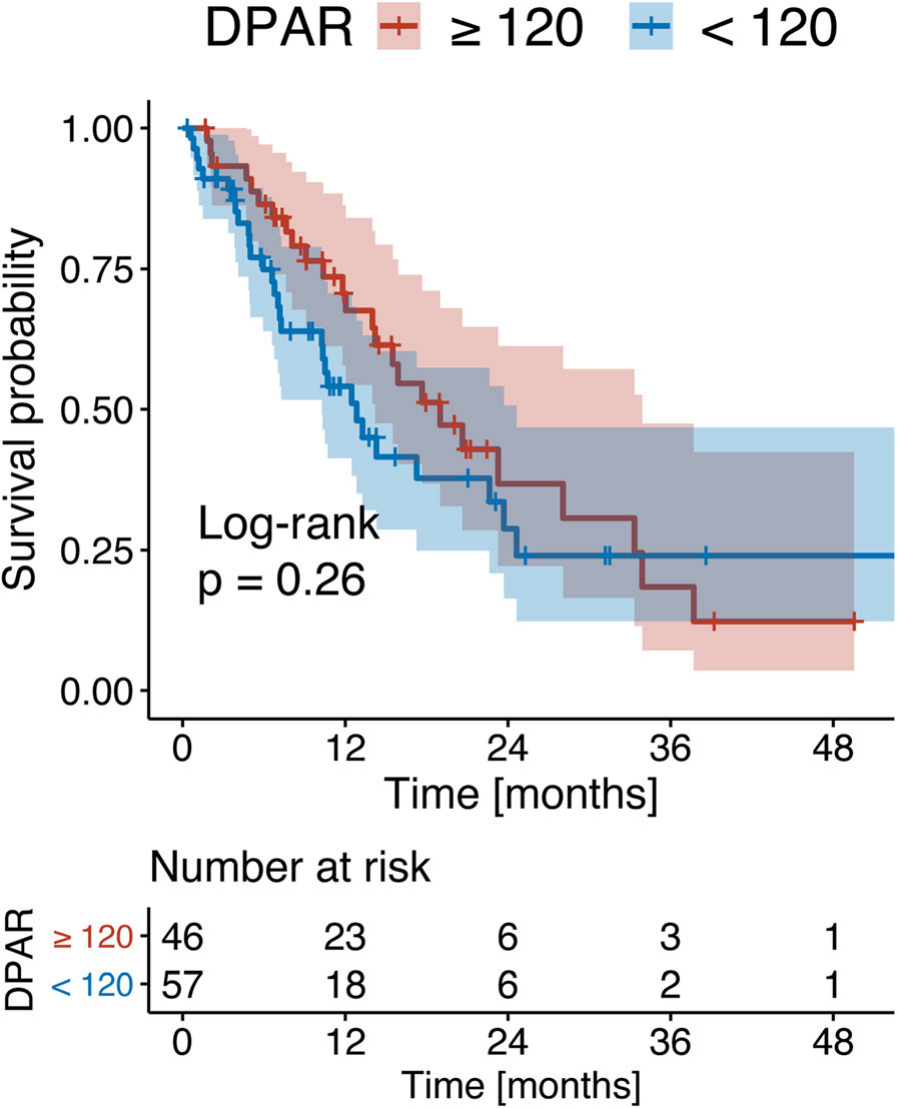
Kaplan Meier curve of the median OS using a DPAR cut-off of 120

Using the previously reported cut-off of 120 for DPAR, patients with a high DPAR also had a higher median OS (18.7 months vs. 12.7 months, Fig. 5). However, no significance was reached (*p* = 0.260). In a second step, we used the optimal cut-off for survival stratification for our cohort (i.e., DPAR of 121), which is very close to the previously reported cut-off. However, no significance was reached (18.7 months vs. 12.7 months, *p* = 0.230).

In a subsequent Cox regression analysis, high albumin, high bilirubin, large tumor size prior to TACE and the BCLC stage were associated with impaired survival (Table 4). The DPAR did not reach significance in the univariate Cox regression analysis. In the subsequent multivariate analysis only a high bilirubin level remained independent prognostic factors.

**Table 4:** Univariate and multivariate Cox regression model with other established risk factors for median OS

Analysis		Univariate			Multivariate		
Covariate		HR	95% CI	P-value	HR	95% CI	P-value
*Age*	*≥ 70 years*	0.9	0.6–1.6	0.800			
*BCLC*	*Stage C or D*	2.1	1.1–4.0	**0.034**	1.4	0.6–3.2	0.384
*AFP*	*> 200 ng/ml*	1.5	0.8–2.6	0.190			
*Albumin level*	*≥ 35 g/l*	2.3	1.2–4.3	**0.010**	1.8	0.9–3.6	0.121
*Bilirubin level*	*≥ 1.2 mg/dl*	2.7	1.5–4.7	**< 0.001**	2.5	1.4–4.5	**0.003**
*AST level*	*> 31 U/L*	2.5	0.6–10	0.210			
*ALT level*	*≥ 35 U/L*	1.0	0.6–1.7	0.980			
*INR*	*> 1.2*	1.0	0.6–1.8	0.910			
*Platelet count*	*< 100/mm*^*3*^	1.3	0.8–2.3	0.270			
*Multifocality*	*Yes*	1.3	0.6–2.6	0.490			
*Tumor size*	*> 5.0 cm*	1.8	1.0–3.1	**0.041**	1.7	0.9–3.2	0.125
*DPAR*	*> 120*	1.4	0.8–2.3	0.260			

AFP, alpha fetoprotein. AST, aspartate aminotransferase. ALT, alanine aminotransferase. BCLC, Barcelona Clinical Liver Cancer. DPAR, delayed percentage attenuation ratio.

## Discussion

In this study, we investigated the role of the novel imaging biomarker DPAR compared to other CT wash-in and washout indices regarding its influence on short-, mid-, and long-term survival outcome after TACE. The average DPAR of patients who survived the first six months after TACE was significantly higher. Furthermore, the rate of patients with a DPAR ≥120 was significantly higher in this group. Thus, DPAR was a prognostic factor for the short-term survival outcome in our study. However, at later time points, DPAR differed not significantly. In addition, DPAR did not significantly predict median OS when using the suggested cut-off of 120 for our cohort. None of the other quantitative washout parameters showed any significance at any investigated time point.

Washout in the venous and delayed phase is the central tumor-defining feature for the non-invasive diagnosis of HCC and, therefore, is part of the Liver Imaging Reporting and Data System used for lesion classification in patients with liver cirrhosis [3, 27, 28]. Thus, multiphasic imaging is the standard diagnostic work-up for suspicious lesions [3]. Delayed phase imaging in particular has high sensitivity for the washout assessment [17, 18]. In clinical routine, the lesion washout is mainly assessed qualitatively as a relative decrease in attenuation from early to later imaging phases. However, several studies have demonstrated the easy and reproducible application of quantitative washout assessment [29, 30].

In a recent study, Fronda et al. were the first to investigate quantitative washout as a predictor of early outcomes after TACE in patients with HCC [19]. They found a significant difference in all investigated wash-in and washout indices between patients with and patients without a complete response to the initial TACE. However, only a DPAR ≥120 remained an independent factor in binary logistic regression. Fronda et al. hypothesized that the superiority of the DPAR may be explained by the independence of the arterial enhancement of the tumor area in calculating the index. This is accompanied by up to one-third of HCCs having no arterial hyperenhancement [15, 16]. Fronda et al. supported their argument with the higher deviation of arterial tumor enhancement compared to the delayed phase in their study [19]. In the present study, the median absolute deviation in the enhancement of the tumor area was also distinctly higher than in the venous or delayed phase, supporting their hypothesis.

Despite its promising power in response and early outcome prediction, our results indicate a decreasing predictive ability of DPAR during the course of disease. In our cohort, only estimates for the remnant liver function and tumor burden were significantly associated with an impaired median OS. This leads us to the hypothesis that, during the course of disease, especially the liver function becomes more important as a treatment-limiting factor. Several studies have previously shown that a worsening of Child-Pugh score and ALBI score after the initial TACE is correlated with an impaired median OS and progression-free survival [31–36]. Furthermore, previous results by Labeur et al. indicate that patients with intrahepatic tumor progression but preserved liver function are more often able to receive further locoregional or systemic treatment beyond TACE, while patients with a worsening of the liver function are “unlikely to benefit from the currently available subsequent treatments” [36]. Therefore, during the course of the disease, the remaining liver function becomes more important in relation to the tumor burden to support treatment-related decision-making.

Nevertheless, in line with Fronda et al.’s assumptions, it may be possible that delayed washout represents the opposite of arterial hypervascularization of the tumor tissue [19]. Thus, the DPAR may be a robust estimate of the technical success of embolization therapies. Several studies have also reported the response to be one of the most important factors for a patient’s prognosis after the initial TACE [31, 33, 37, 38]. Therefore, the combination of DPAR with the remnant liver function prior and after the initial TACE, as well as the remaining tumor burden, could be superior in prognosis modelling and serve as a basis for future studies. As Fronda et al. mentioned, this could be particularly important for the application of artificial intelligence studies in HCC, as they are mainly based on arterial phase imaging [19, 39, 40].

Interestingly, the optimal DPAR cut-off of 121 for survival prediction in our cohort was very close to the cut-off of 120 reported by Fronda et al. [19]. In general, studies on DPAR as a diagnostic or novel prognostic parameter are scarce. Thus, large prospective trials are needed to further validate the usefulness of the DPAR in patients with HCC and to define reliable cut-off values for its use in daily clinical routine, especially because quantitative washout assessment is easy to apply in clinical routine without any additional diagnostic effort and without additional costs.

In this study a tube voltage of 120 kVp was used for the acquisition of the delayed phase scans. The same tube voltage was used in the original study on the DPAR by Fronda et al. [19]. Thus, DPAR has only been validated for a tube voltage of 120 kVp, so far. While a tube voltage of 120 kVp has been standard in several studies on washout indices in patients with HCC [16–18], other authors argue for a use of a lower tube voltage, not only for arterial phase scanning but also for venous and delayed phase scans [41]. Given the differences in contrast to noise ratio, it remains unclear how robust the DPAR is in case of other tube voltages. Therefore, we encourage other groups to investigate the reproducibility and robustness of the DPAR for other tube voltages in future studies.

The present study has several limitations. First, this study was conducted as a single center study. Second, the sample size was only moderate (*n* = 103). However, the sample size was comparable to other studies on this issue [19]. One limiting factor for the sample size may be the decision against imputing missing values. Only patients with complete datasets were included in order to maintain high data quality. Furthermore, only patients from 2016 onwards were included to guarantee the comparability of pre-procedural imaging, the procedure itself, and follow-up. In addition, only patients without any previous treatment were included. Third, we excluded patients who underwent subsequent liver transplantation or other curative treatments after TACE in order to avoid bias [42]. Fourth, DPAR was calculated based on the delayed phase tumor attenuation assessed in a single ROI in the largest tumor area as suggested by Fronda et al. [19]. However, two-dimensional assessment of the tumor attenuation might underestimate the heterogeneity of the tumor tissue. Nevertheless, our supplementary analysis regarding the attenuation in various tumor regions of the largest TL showed that especially the delayed phase showed a high correlation between the attenuation in various tumor regions. Fifth, we did not perform any subgroup analyses of patients treated with different TACE techniques because multiple previous comparisons between cTACE and DEB-TACE have not shown any influence on OS [43–45].

## Conclusion

Our results confirm the value of the DPAR as the most relevant washout index for predicting the short-term outcome in patients with HCC undergoing TACE. However, the DPAR and other washout indices did not reach significance in predicting the mid- and long-term outcome. Thus, factors associated with the patient’s remnant liver function and tumor burden may become more important during the disease course. However, as a novel and innovative risk factor, the DPAR may contribute to improved longitudinal prognosis modelling in combination with various other prognostic factors, which should be evaluated in future prospective trials.

## Supplementary Information

**Additional file 1.**

## Data Availability

Data cannot be shared publicly because of institutional and national data policy restrictions im-posed by the Ethics committee of the Medical Association of Rhineland Palatinate, Mainz, Ger-many since the data contain potentially identifying patient information. Data are available upon request for researchers who meet the criteria for access to confidential data.

## Abbreviations

AFP: Alpha fetoprotein
AASLD: American Association for the Study of Liver Diseases
ALT: Alanine aminotransferase
AST: Aspartate aminotransferase
AIH: Autoimmune hepatitis
BCLC: Barcelona Clinic Liver Cancer
CRU: Clinical registry unit
CR: Complete response
CT: Computed tomography
CIs: Confidence intervals
cTACE: Conventional transarterial chemoembolization
DPAR: Delayed percentage attenuation ratio
DEB-TACE: Drug-eluting bead transarterial chemoembolization
EASL: European Association for the Study of the Liver
HRs: Hazard ratios
HCC: Hepatocellular carcinoma
HU: Hounsefield unit
kVp: Kilovoltage peak
Q1: Lower quartile
MAD: Median absolute deviation
mA: Milliampere
NASH: Nonalcoholic steatohepatitis
OS: Overall survival
PR: Partial response
PBC: Primary biliary cholangitis
PSC: Primary sclerosing cholangitis
PD: Progressive disease
ROI: Region of interest
TLs: Target lesions
SD: Stable disease
SD: Standard deviation
TACE: Transarterial chemoembolization
Q3: Upper quartile
