# Supplementary material for: Quantitative washout in patients with hepatocellular carcinoma undergoing TACE: an imaging biomarker for predicting prognosis?
Source: Cancer Imaging. 2022 Jan 11;22:5. doi: 10.1186/s40644-022-00446-6 (PMC8753936; doi:10.1186/s40644-022-00446-6)
Supplement: Supplementary file 1 — Additional file 1. [file 40644_2022_446_MOESM1_ESM.docx]

**Supplementary Figure 1:** Correlation of the attenuation of the largest tumor region and the upper/lower tumor region in the arterial phase (A, B), the venous phase (C, D) and the delayed phase (E, F)
